# Synthetic computed tomography for low-field magnetic resonance-guided radiotherapy in the abdomen

**DOI:** 10.1016/j.phro.2022.11.011

**Published:** 2022-11-28

**Authors:** Mariia Lapaeva, Agustina La Greca Saint-Esteven, Philipp Wallimann, Manuel Günther, Ender Konukoglu, Nicolaus Andratschke, Matthias Guckenberger, Stephanie Tanadini-Lang, Riccardo Dal Bello

**Affiliations:** a Department of Radiation Oncology, University Hospital Zurich and University of Zurich, Zurich, Switzerland; b Artificial Intelligence and Machine Learning Group, Department of Informatics, University of Zurich, Zurich, Switzerland; c Computer Vision Laboratory, ETH Zurich, Zurich, Switzerland

**Keywords:** MR-only radiotherapy, Synthetic CT, MR-Linac, CycleGAN, Neural network

## Abstract

**Background and purpose:**

The requirement of computed tomography (CT) for radiotherapy planning may be bypassed by synthetic CT (sCT) generated from magnetic resonance (MR), which has recently led to the clinical introduction of MR-only radiotherapy for specific sites. Further developments are required for abdominal sCT, mostly due to the presence of mobile air pockets affecting the dose calculation. In this study we aimed to overcome this limitation for abdominal sCT at a low field (0.35 T) hybrid MR-Linac.

**Materials and methods:**

A retrospective analysis was conducted enrolling 168 patients corresponding to 215 MR-CT pairs. After the exclusion criteria, 152 volumetric images were used to train the cycle-consistent generative adversarial network (CycleGAN) and 34 to test the sCT. Image similarity metrics and dose recalculation analysis were performed.

**Results:**

The generated sCT faithfully reproduced the original CT and the location of the air pockets agreed with the MR scan. The dose calculation did not require manual bulk density overrides and the mean deviations of the dose-volume histogram dosimetric points were within 1 % of the CT, without any outlier above 2 %. The mean gamma passing rates were above 99 % for the 2 %/ 2 mm analysis and no cases below 95 % were observed.

**Conclusions:**

This study presented the implementation of CycleGAN to perform sCT generation in the abdominal region for a low field hybrid MR-Linac. The sCT was shown to correctly allocate the electron density for the mobile air pockets and the dosimetric analysis demonstrated the potential for future implementation of MR-only radiotherapy in the abdomen.

## Highlights

• This study investigated the generation of synthetic computed tomography for magnetic resonance only radiotherapy in the abdominal area.
• The network cycle-consistent generative adversarial network was used, which can be trained on unpaired data.
• The allocation of Hounsfield units for mobile air pockets in the synthetic computed tomography were in agreement with the magnetic resonance.
• The dosimetric comparison between the synthetic and planning computed tomography showed excellent agreement.

## 1 Introduction

The introduction of magnetic resonance (MR) guided radiotherapy (MRgRT) systems into clinical practice brought personalised medicine one step forward. The coupling of a linear accelerator (Linac) with the most versatile imaging modality (MR) provides unique opportunities to radiotherapy (RT) [1], [2], [3]. The on-board MR scanner allows to position the patient with high accuracy and the good soft tissue contrast makes plan adaptation to the daily anatomy possible [4]. Treatment gating based on internal organ motion is made possible with real-time MR imaging during RT delivery [5], [6]. While all the previous features rely solely on the MR modality, it is still necessary to perform an additional computed tomography (CT) simulation for treatment plan preparation and dose calculation [7]. Removing the latter requirement would allow the transition to MR-only radiotherapy [8].

MR-only radiotherapy brings additional benefits including the elimination of registration uncertainties between CT and MR, a reduction in radiation exposure and a more efficient and cost-effective workflow [8]. The CT is used to compute the electron density (ED) map, required for dose calculation. Currently implemented clinical workflows perform the MR simulation at the MR-linac itself followed by a CT scan [9], [10], [11]. The latter could be substituted by a synthetic CT (sCT), which may be generated by different methods such as tissue segmentation-based, learning-based and atlas-based approaches. Han demonstrated the superiority of deep learning (DL) for this task [12]. Several investigations followed, aiming to generate sCT from MR imaging for RT applications [13] for several sites [14], [15], [16]. Dosimetric deviations between CT and sCT generated with DL are generally lower than 1 % [13], which satisfies the 2 % requirement for clinical applicability [17].

Commercial solutions are available for sCT generation for head and neck (MRCAT, Philips, Eindhoven, The Netherlands), prostate and brain (syngo.via, Siemens, Erlangen, Germany). On the other hand, it has been identified that further developments are needed for sCT to pelvic and abdomen sites other than prostate [18]. One of the main limitations being the presence of mobile air pockets. Their different location in MR and CT requires either a manual override of the ED [19] or the manual selection of a subset of patients with similar air pockets distribution in MR and CT to train networks requiring paired images [20]. An elegant solution is provided by CycleGAN, which introduces a cycle consistency, ensuring that the sCT can be back-transformed into a synthetic MR matching the original image [21]. This additional back-transformation increases the computational burden but allows training the network on unpaired data, *i.e.* the MR and CT can potentially be acquired from different patients. Substantial performance improvements can be achieved if MR and CT have some degree of correlation, imaging similar anatomies with the two different modalities [21], which can be achieved by training the network with dual-domain unpaired data [22] from the same subjects.

A recent review highlighted the need of further studies confirming the preliminary results and cover all sites and magnetic field strengths [13]. The abdomen was analysed only marginally [20], [23], [24], [25], [26] and the vast majority (94.5 %) of results were reported from MR images acquired with 1.5 T or higher fields. To date, a few additional investigations were conducted with fields below 1 T [27], [28], [29]. The sCT generation task in the abdomen at low field was investigated only in four studies with U-Net [28], Pix2pix [20] and a combination of multiple networks including CycleGAN [24], [30]. The common limitation is the number of patients to train and test the network in the abdomen (respectively: 37, 60, 12, 30). In the current study we aim to overcome this limitation and focus exclusively on the abdomen area. Moreover, we address the problem of mobile air pockets by generating sCT with networks trained on unpaired data.

## 2 Materials and methods

### 2.1 Dataset

This study retrospectively analysed 215 MR-CT pairs from 168 patients treated at the University Hospital Zurich in the period August 2020 - May 2022 (Supplementary Table S1). We excluded 29 MR-CT pairs with implants or contrast agents. No additional exclusion criteria were applied based on image quality or anatomy to minimise the requirement of manual intervention and generalise the model. The remaining 186 MR-CT were further stratified into patient-separated train and test sets with a 80 %–20 % ratio. Keeping the proportionality among the treatment sub-sites, it led to 152 and 34 MR-CT pairs in each group, respectively. All institutional guidelines were followed. Informed consent was obtained from all patients. All patients gave their consent for retrospective data analysis. The study was approved by the cantonal ethics committee Zurich (BASEC-Nr. 2018-01794).

### 2.2 Image acquisition and pre-processing

The MR images were acquired with a true fast imaging with steady-state precession (TrueFISP) sequence at the 0.35 T MRI scanner of the MRIdian system (ViewRay, Ohio, USA). The axial resolution was 3 mm and the planar resolution ranged from 1.49 to 1.63 mm depending on the field of view (FOV). The axial direction covered 24 cm (80 slices) and the scans were acquired in expiration breath hold (EBH). The data included MR images acquired both before and after several major upgrades (MRIdian software software v5.3.1, Smart VISION in March 2021 and MRIdian hardware system v2.0, High Speed MLC in May 2021) of the imaging system, the receiver coils and gantry geometry, which are known to affect the imaging performances [31].

The CT images were acquired typically within 60 min after the MR, adopting the same immobilisation setup and using EBH. The scans were performed with a Somatom Definition AS (Siemens, Erlangen, Germany) operated at 120 kVp. The voxel resolution was equal or superior to the MR resolution.

The CT images were imported to the MRIdian TPS and registered to the MR (dCT) with deformable registration. The registration was performed with the proprietary algorithm implemented in the MRIdian TPS with default settings [32], [33]. The registrations were reviewed and approved by an experienced radiation oncologist inspecting the location and deformation of organs at risk (OAR) and targets. Additionally, a medical physicist reviewed the registrations verifying the suitability of the FOV for the adaptive workflow and the location and deformation of hyper- and hypo-dense regions affecting the dose calculation. The MR and dCT were resampled to the modal resolution 3x1.63x1.63 cm^3^ and cropped to volumes of 40x256x256 voxels. This covered 12 cm in the axial direction including the PTV and 2 cm margin but excluding the edges of the FOV not relevant for the dose calculation. The dCT intensity values were clipped to the HU range from air to cortical bone (1200), and scaled to the range [0,1]. The MR data was normalised with the piecewise histogram-based technique proposed by Nyul *et al.* [34] and implemented by Reinhold *et al.* [35]. The outliers in the upper and lower 2^nd^ percentiles were clipped within the output range [0,1]. This is referred to as *Nyul normalisation*.

### 2.3 Network architecture

The pre-processed MR and dCT were used to train CycleGAN [21] with dual-domain unpaired data from the same subjects, which is referred to as *unpaired data* in this study. The MR and dCT contained similar but not identical anatomical features due to the physiological changes between the MR and CT scans. The specific implementation [36] was performed with ResNet-based generators and PatchGAN discriminators. The following cost function was used to optimise the network:

$$\arg\;\min_{G}\max_{D}L_{\mathrm{Gan}}\left(G_{\mathrm{MR}}^{\mathrm{CT}},G_{\mathrm{CT}}^{\mathrm{MR}},D_{\mathrm{MR}},D_{\mathrm{CT}}\right)+\lambda_{\mathrm{cyc}}L_{\mathrm{cyc}}\left(G_{\mathrm{MR}}^{\mathrm{CT}},G_{\mathrm{CT}}^{\mathrm{MR}}\right)+\lambda_{\mathrm{identitiy}}L_{\mathrm{identity}}\left(G_{\mathrm{MR}}^{\mathrm{CT}}G_{\mathrm{CT}}^{\mathrm{MR}}\right),$$

where $G_{\mathrm{CT}}^{\mathrm{MR}}$ represents the generator in the direction of MR → CT, $D_{\mathrm{CT}}$ the discriminator between dCT and sCT images, $\lambda_{\mathrm{cyc}}$ and $\lambda_{\mathrm{identity}}$, are the weight factors for the cycle and the identity losses, which were set equal to 10 and 5, respectively [21]. We highlight the importance of the cycle-consistency: not only the MR was used to generate an sCT, but also the sCT was back-transformed into a synthetic MR. The same process was applied to the dCT. The cycle-consistency was ensured by minimising the L1 distance between the synthetic and the original MR or dCT. The introduction of two generators with the aim of reproducing the original image is key to removing the requirement on paired MR-dCT images to train the network.

### 2.4 Training

The network training was performed on a high performance cluster with Nvidia GeForce 1080 Ti (11 GB RAM) GPU and 50 GB of allocated memory. The network was operated in 2D, passing axial slices to the single-channel input and output. Based on the fine-tuning results, the hyperparameters were: GAN mode LSGAN, batch size 1, size of image buffer 80, Adam optimizer, learning rate 0.0002 and number of epochs 100.

### 2.5 Evaluation

The similarity between real and synthetic CT images was evaluated with the following quantitative evaluation metrics within the body contour: mean absolute error (MAE), mean squared error (MSE), peak signal-to-noise ratio (PSNR), structural similarity index (SSIM) and Frechet-Dirichlet distance (FID) [13], [37]. Then, the sCT were imported into the MRIdian TPS. No registrations were necessary since the network provided sCT aligned to the input MR. The sCT were converted to electron density map with the same Hounsfield unit lookup table used for the dCT, but without any bulk override structure [33]. Note that dCT and MR present air pockets in different locations and manual contouring was performed on the MR to assign a density of 1.2⋅10^-3^ g/cm^3^ to such voxels during dose calculation. The plans originally prepared on the dCT (dose dD) were rigidly copied and recalculated on the sCT (dose sD) with the same number of monitor units using the following Monte Carlo setting: grid size 0.2 cm, magnetic field corrections activated, variance 1 %.

The dose-volume histogram (DVH) comparison included the target coverage (PTV D95%), near-maximum (PTV D2%), near-minimum (PTV D98%) and mean dose (PTV Dmean and GTV Dmean) [38]. The OAR were evaluated through the near-maximum dose (Stomach D2%, Duodenum D2%, Bowel D2%, Spine D2%) and the mean dose (Ipsilateral Kidney Dmean, Liver Dmean, Heart Dmean). We also evaluated the mean dose to ring structures up to 2 cm and 4 cm from the PTV (Ring2 Dmean, Ring4 Dmean). The dosimetric points calculated on the sCT (sP) were compared to their value on the dCT (dP). We performed a Z-test with known variance from the Monte Carlo dose engine to assess whether the differences between sP and dP were significant (p < 0.05). The dose matrices were evaluated through a 3D local gamma analysis with passing criteria 1 % / 1mm or 2 % / 2 mm and two dose thresholds: within the volume receiving 90 % and 50 % of the prescribed dose.

## 3 Results

### 3.1 Image comparison between sCT and dCT

**Fig. 1:**
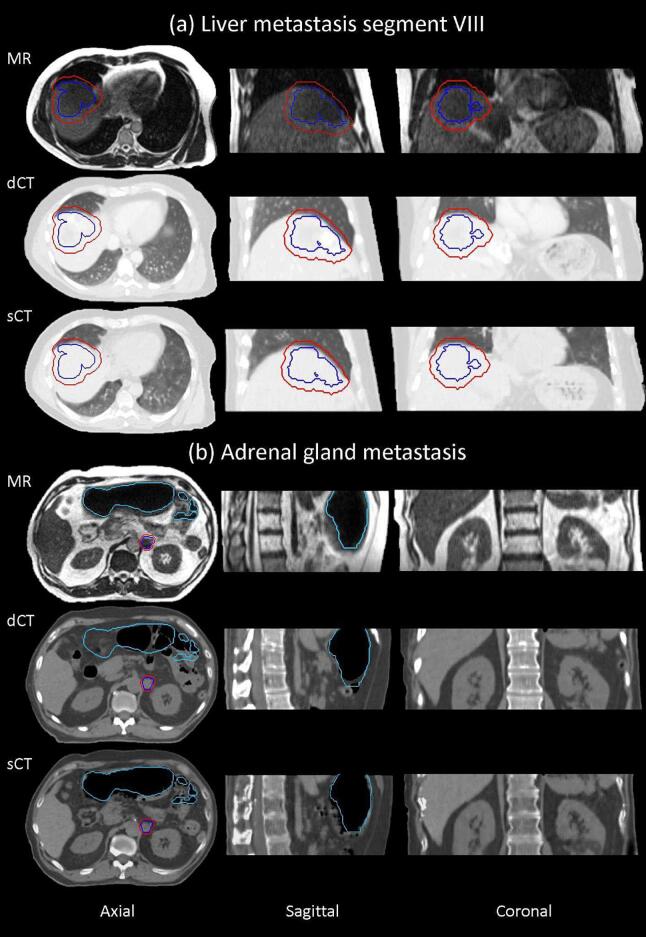
Example of sCT for a liver case (a) displayed in the lung CT window and an adrenal gland case (b) in soft tissue window. The contours of the PTV (red), GTV (blue) and air pockets in the MR image (cyan) are also displayed. The axial slices are located at the centre of the PTV (a, b) and the coronal and sagittal either at the centre of the PTV to show the lung tissue (a) or the vertebral bodies to show the bones (b). (For interpretation of the references to colour in this figure legend, the reader is referred to the web version of this article.)

**Fig. 2:**
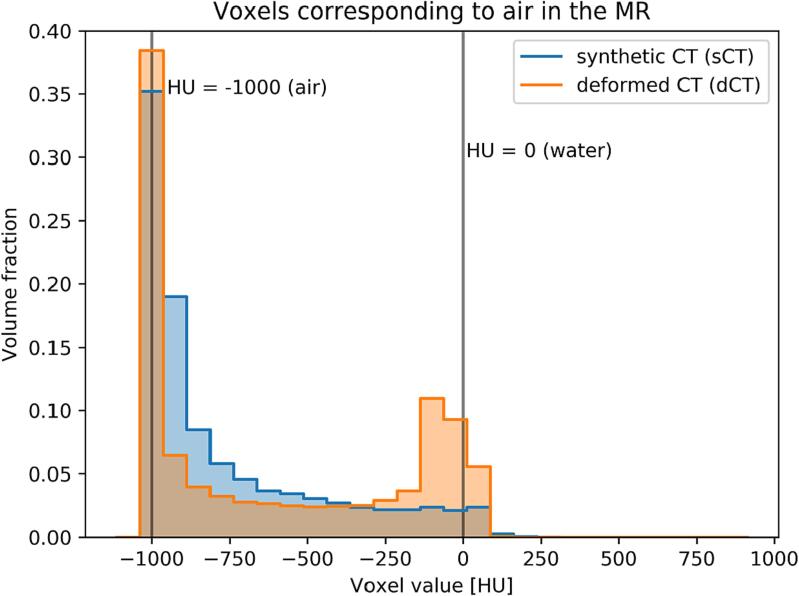
Distribution of the voxel values in sCT and dCT with the air contour delineated on the MR image. The histograms cover the whole test cohort. The HU nominal values of air and water are reported as reference. The main peak at low HU corresponding to the air pockets is observable in both histograms. For the sCT, a tail extends to higher HU values whereas for the dCT, a second peak corresponding to soft tissue is observable.

The trained network generates single sCT slices within 15 s. Two representative cases are shown in Fig. 1. The qualitative analysis shows that the reproduction of soft tissue, lung and vertebral bodies in the sCT is comparable to the dCT. The individual vertebral bodies are clearly visible in all axial, coronal and sagittal views (Fig. 1b). While the ribs have a sharp contour in the axial view, this feature is lost in the coronal slices. The location of the air pockets is better aligned to the MR in the sCT than in the dCT that was taken up to 60 min after the MR. The Dice-coefficient between the air manually contoured on the MR and a thresholding automatic contouring (HU <−500) on the CT improves from 0.40 ± 0.19 (dCT) to 0.65 ± 0.17 (sCT). The quantitative image comparison is reported in Table 1. Fig. 2 shows the distribution of the voxel values in sCT and dCT and the air is delineated on the MR images. Both histograms present the main peak at low HU corresponding to the air pockets. While only a tail extends to higher values in the sCT, a second peak corresponding to soft tissue is observable for the dCT. Smaller differences are observed for the soft tissue and bone histograms (Supplementary Figure S1 and S2),

**Table 1:** Quantitative analysis of the image similarity between sCT and dCT. The parameters are calculated within the body mask.

Metric	Mean ± SD
MAE	70.10 ± 18.97
MSE	2158 ± 529
PSNR	39.02 ± 1.00
SSIM	0.981 ± 0.009
FID	21.41

### 3.2 Dosimetric comparison between sCT and dCT

**Fig. 3:**
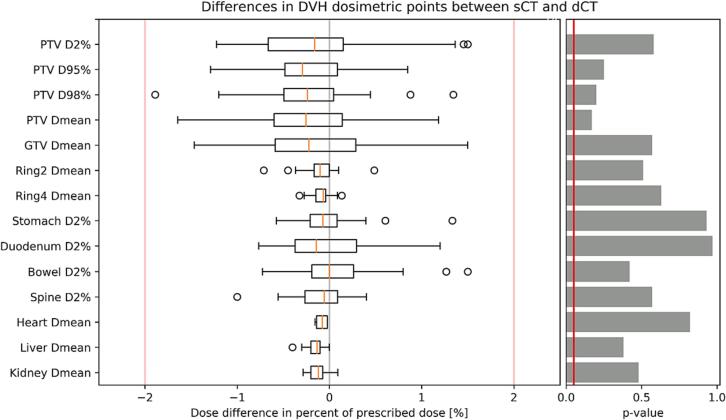
Differences in DVH dosimetric points between plans calculated on the sCT and dCT. Vertical lines provide an aid to identify the reference at 0 % (grey) and the ± 2 % limits (red). The right panel reports the results of the Z-test. The significance level p = 0.05 is highlighted with a vertical line and no values below it are observed. (For interpretation of the references to colour in this figure legend, the reader is referred to the web version of this article.)

The recalculation of the plans on the sCT shows excellent agreement with the dCT. The summary is reported in Fig. 3. The mean of the differences are below 1 % of the prescribed dose (below 0.5 Gy for absolute differences). No deviations above 2 % (1 Gy) and no significant difference between sP and dP are observed.

**Fig. 4:**
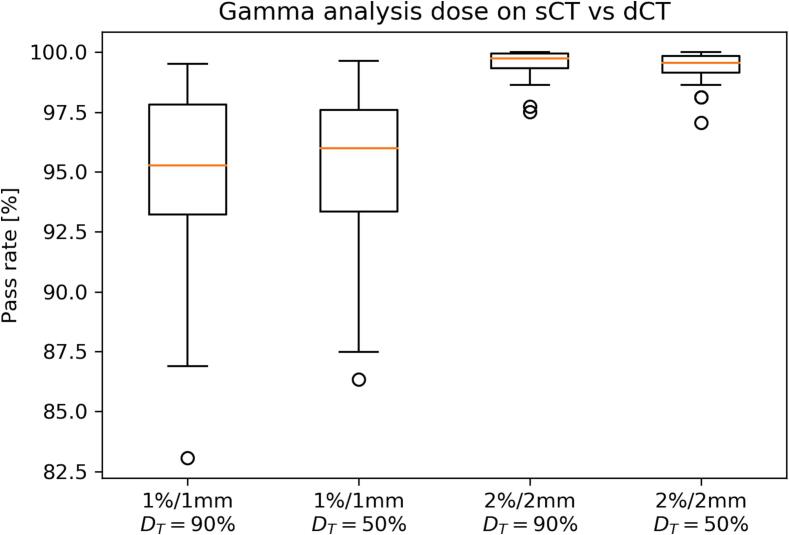
Boxplots of the gamma pass rate distribution for the analysis performed with 1 %/1mm and 2 %/2 mm criteria. The dose thresholds are set to 90 % and 50 % of the prescribed dose. The mean pass rates are: 94.9 ± 3.7 % (1 %/1mm, D_T_ = 90 %), 95.1 ± 3.3 % (1 %/1 mm, D_T_ = 50 %), 99.5 ± 0.6 % (2 %/2 mm, D_T_ = 90 %) and 99.4 ± 0.6 % (2 %/2 mm, D_T_ = 50 %).

**Fig. 5:**
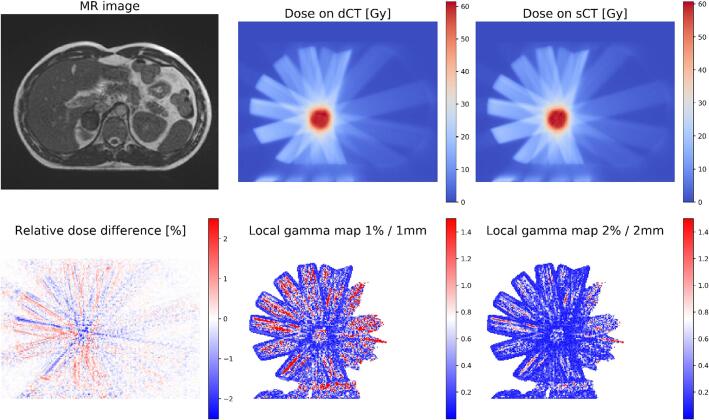
Example of dose calculation on dCT (centre top) and sCT (right top). The analysis of the voxel-by-voxel difference (left bottom) and gamma analysis with 1 %/1mm (centre bottom) and 2 %/2 mm (right bottom) criteria are reported along with the MR image (left top). The gamma pass rates in this specific example were 94.8 % (1 %/1 mm) and 99.4 % (2 %/2 mm) for the analysis with D_T_ = 50 %.

The gamma analysis confirms the agreement between the dose matrices. The mean passing rates for the 1 %/1mm analysis with thresholds at 90 % and 50 % are 94.9 ± 3.7 % and 95.1 ± 3.3 %, respectively. The 2 %/2mm analysis has mean passing rates above 99 %, without any case below 95 %. Their distributions are reported with boxplots in Fig. 4. An example of dose recalculation and analysis is shown in Fig. 5. The analysis for the two outliers in the 1 % / 1 mm gamma analysis is reported in the supplementary material (Supplementary Figures S4 and S5). The DVH of all the patients presented are reported in the supplementary material (Supplementary Figures S6–S10).

## 4 Discussion

The present study aims to improve the state-of-the-art sCT generation in the abdomen region for enabling the transition to MR-only radiotherapy. Previous investigations demonstrated the application of DL methods but faced limitations due to the patient number [24], [26] or networks relying on perfectly aligned images [20]. The current work overcomes the former by focusing exclusively on the abdomen increasing the patient number and the latter by training CycleGAN on unpaired data.

The high patient number in the training set provides a reliable generation of sCT, including cases where the GTV extends to the cranial part of the liver and dose calculation through lung tissue is required (Fig. 1a). The MAE shows a monotonic behaviour with increasing patient number, improving from MAE = 89.8 with 12 patients [24] to MAE = 78.71 with 60 [20] and MAE = 70.10 in the current study. One study is excluded due to a different MAE calculation approach [28]. The monotonic improvement confirms and extends previous results limited to a smaller number of patients [27]. The bone reproduction quality also improves (Fig. 1b). The sharpness of vertebral bodies and location of ribs improved compared to networks trained on fewer patients. The quantitative comparison of the voxels values within the vertebral body contour is reported in the supplementary materials (Supplementary Figure S2). The loss of rib sharpness in the coronal view can be attributed to the 2D design of the network. A pseudo-3D approach was demonstrated to improve the bony anatomy reproduction in the brain [39] and should be considered for future investigations with the current data set. It is important, however, to notice that the patient positioning at the MR-Linac does not rely on kV imaging. The need for bone contrast in the digitally reconstructed radiograph (DRR) is replaced by the daily MR to MR registration [40].

The air pockets are relevant for dose calculation [19]. While an exact reproduction of the air HU is critical for dose calculation in proton beam therapy [41], photon treatments are more robust and are mostly influenced if the air is in close proximity or within the PTV [42]. The two outliers in the 1 %/1mm gamma analysis in Fig. 4 are patients in which multiple beams cross an air pocket within a few centimetres before reaching the PTV. The dCT is corrected with a bulk override over the whole air pocket, while the sCT reproduces both air and air-fluid mixtures. This can lead to negative (Supplementary Figure S4) or positive (Supplementary Figure S5) dose deviations. Nonetheless, the OAR DVHs (Supplementary Figures S7 and S9) are unaffected and the target dose deviations are within 2 %. Therefore, the sCT and dCT are both suitable for dose calculation, but the presence of manual bulk overrides in the dCT introduces greater variability compared to the sCT. The sCT generation with CycleGAN provides a reliable reproduction of the air pockets from the MR removing the requirement of manual bulk density override, which is instead required for dose calculation on dCT (Fig. 2). We attribute this to the training of the network on unpaired images. While the simulation MR and CT are acquired within a short time frame, anatomical differences are still present. Networks such as Pix2pix requiring perfectly aligned image pairs cannot be expected to converge with the same accuracy as CycleGAN under such settings [21].

The dosimetric comparison between dCT and sCT shows an excellent agreement within the clinically acceptable range [17]. The dose differences are on average below 1 % for all the DVH dosimetric points and no deviations above 2 % are observed. The absence of outliers can be interpreted by the introduction of a statistical normalisation technique in the MR pre-processing, which was previously reported as beneficial in standardising MR datasets before machine learning processing [43]. Despite using one unique scanner, the image quality can have intra-day variations and several major upgrades were performed within the study. Applying MR normalisation can compensate for these differences. The comparison for networks trained and tested independently with and without Nyul normalisation during the pre-processing (Supplementary Table S2 and Supplementary Figure S3) shows no significant variation in the mean deviations. However, the range of the differences is wider in absence of normalisation with larger standard deviations and a few DVH outliers above 2 %. We can conclude that the use of MR normalisation does not increase accuracy, but it increases precision avoiding outliers due to intra-day changes in the MR scanner performances and it is, therefore, preferable.

This study focused and is therefore limited to abdominal treatments. The results cannot be generalised to other treatment sites, which require additional investigations [13]. The dosimetric evaluation is specific to IMRT photon treatments and should not be applied to proton treatments, known to be more sensitive to dCT-sCT differences along the beam path [41]. This is also a single-centre and single-scanner study. Additional investigations are required to assess whether the results are transferable to other MR-Linac. Finally, while the dosimetry is within the clinically acceptable range, the implementation of an independent method to perform quality assurance of the sCT is fundamental for clinical applications and was not part of this investigation.

To conclude, this study improved the state-of-the-art for the implementation of CycleGAN to perform sCT generation in the abdominal region. The dosimetric requirements for a potential application in MR-only radiotherapy were excellent, with deviations from the dose calculated on the planning CT in average below 1 % and without any outliers above 2 %.

## Declaration of Competing Interest

The authors declare the following financial interests/personal relationships which may be considered as potential competing interests: Part of this work was supported by a research grant from ViewRay Inc. (MASPAC study), within the Clinical Research Priority Programme “Artificial intelligence in Oncological Imaging” of the University of Zurich, and SNF R’Equip program (grant 326030_177080/1).
