# Supplementary material for: Synthetic computed tomography for low-field magnetic resonance-guided radiotherapy in the abdomen
Source: Phys Imaging Radiat Oncol. 2022 Nov 28;24:173–9. doi: 10.1016/j.phro.2022.11.011 (PMC9720490; doi:10.1016/j.phro.2022.11.011)
Supplement: Supplementary data 1 [file mmc1.pdf]

Supplementary material

	Patient cohort before exclusion criteria	Patient cohort after exclusion criteria
Total patient number	168	144
MR-CT pairs (3D images)	215	186
MR-CT pairs (slices)	8’600	7’440
MR-CT with high density in FoV (3D images)	29	0
Treatment site (3D images)		
Liver	82	69
Nodes	59	54
Adrenal glands	30	30
Pancreas	29	20
Kidney	13	11
Spleen	2	2
Male / Female	101 / 67	89 / 55
Mean PTV volume (range) [cm^3]	n.a.	53.9 (6.4 ÷ 291.6)

Supplementary Table S1. Patient characteristics and treatment sites before and after the exclusion criteria. The number of MR-CT pairs is higher than the number of patients because some patients received multiple courses of treatment. High density material includes metal implants, catheters and contrast agents. The PTV volume is based on the test subset.

Metric	No normalisation during pre-processing<br><br>Mean ± SD	Nyul normalisation during pre-processing<br><br>Mean ± SD
MAE	71.20 ± 21.36	70.10 ± 18.97
MSE	2059 ± 642	2158 ± 529
PSNR	39.29 ± 1.24	39.02 ± 1.00
SSIM	0.980±0.010	0.981±0.009
FID	24.53	21.41

Supplementary Table S2. Quantitative analysis of the image similarity between sCT and dCT. The results for a network trained and tested on MR images normalised with the *Nyul normalisation* approach are compared to an independent network trained and tested on MR images without normalisation during the pre-processing. The mean values of the analysed metrics are comparable, however the introduction of normalisation reduces the variability achieving smaller standard deviations.

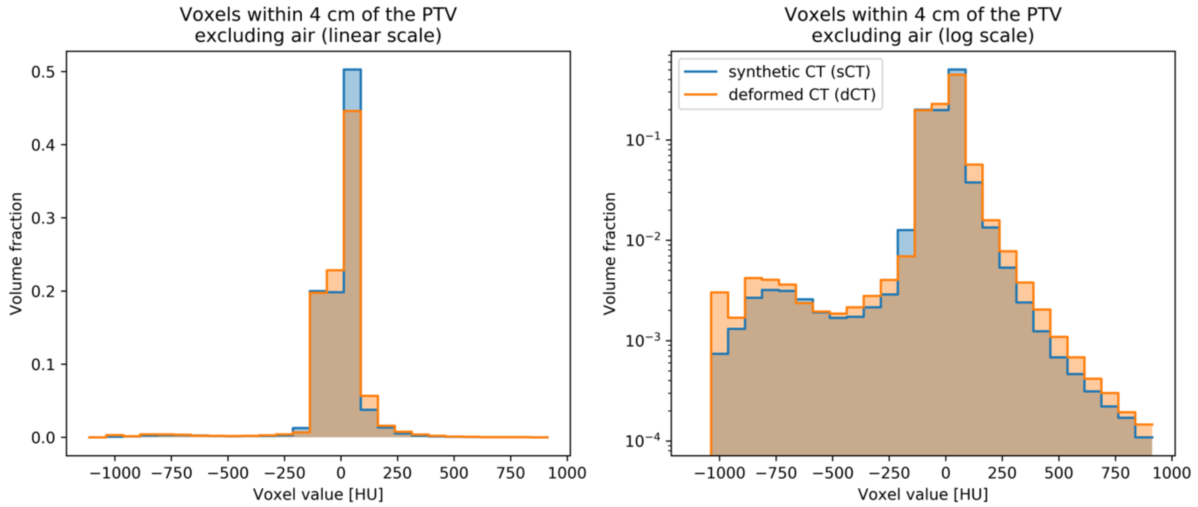

Supplementary Figure S1. Distribution of the voxel values in the sCT and dCT within 4 cm from the PTV. The voxels within the lung and air contours delineated on the MR are removed from the histograms. Left: linear scale in voxel count. Right: logarithmic scale.

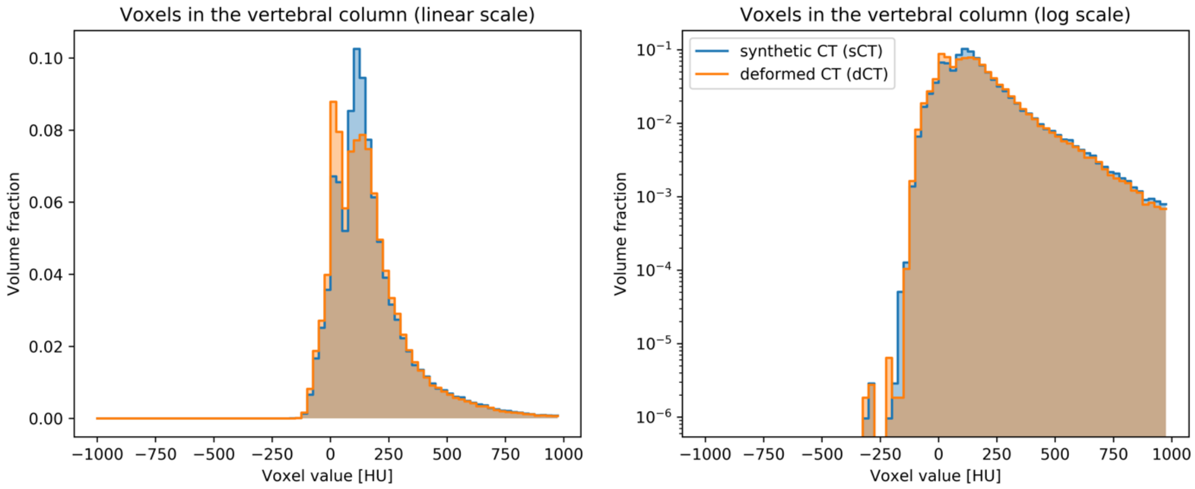

Supplementary Figure S2. Distribution of the voxel values in the sCT and dCT within the vertebral column contour. Left: linear scale in voxel count. Right: logarithmic scale.

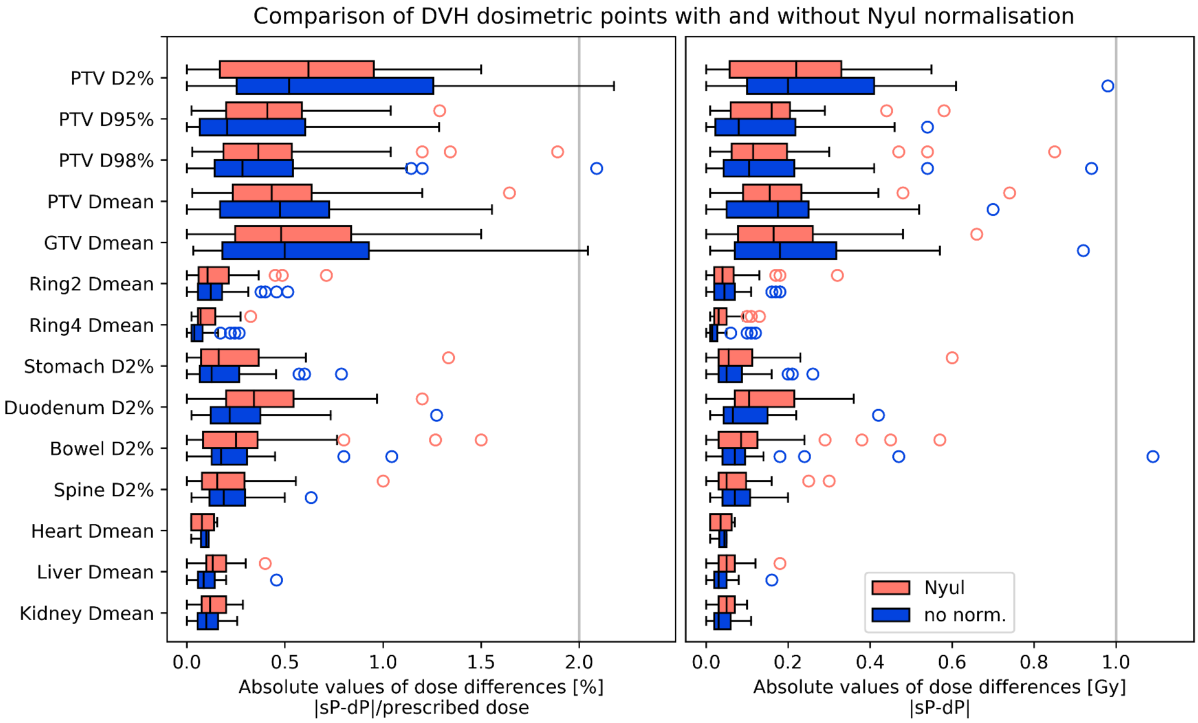

Supplementary Figure S3. Absolute values of the differences in DVH dosimetric points between plans calculated on the sCT and dCT. The comparison between the Nyul normalisation (red) and no normalisation (blue) approach is shown. The differences relative to the prescribed dose (left) are reported along with the absolute differences (right). Vertical lines indicating the threshold of 2% and 1 Gy deviations are reported.

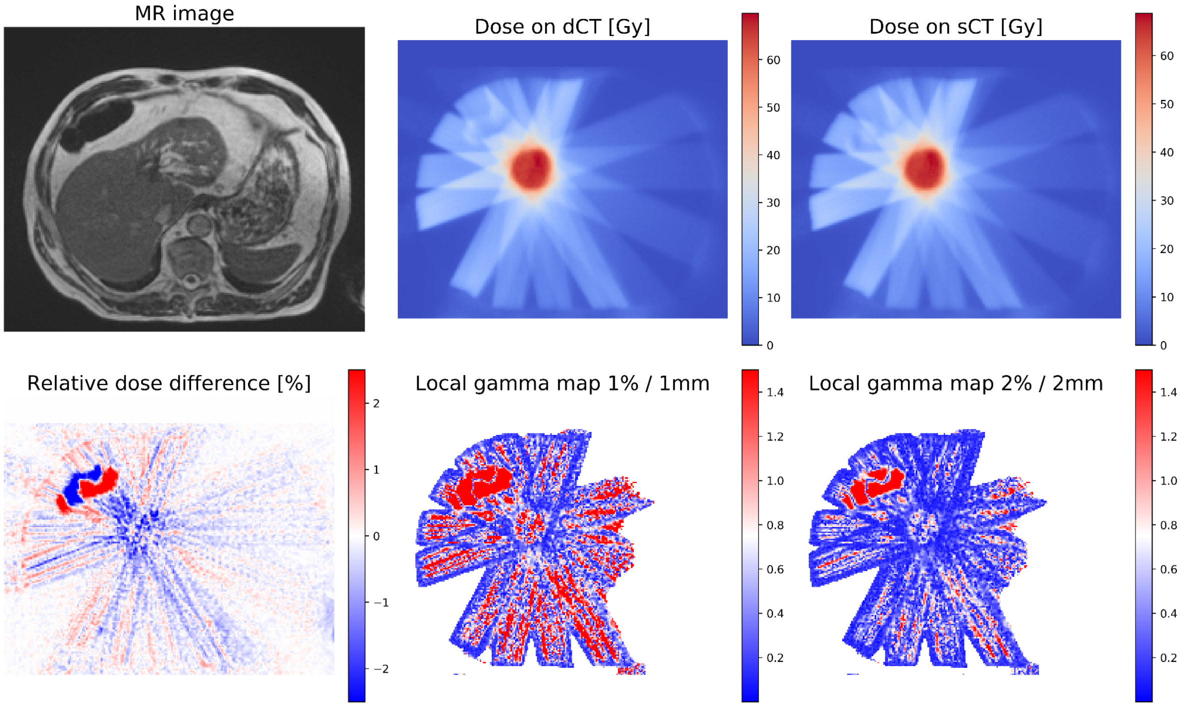

Supplementary Figure S4. Dose analysis for the outlier in the 1%/1mm gamma analysis with 50% threshold in Figure 3. The MR image (left top) is shown along with the dCT (centre top) and sCT (right top). The analysis of the voxel-by-voxel difference (left bottom) and gamma analysis with 1%/1mm (centre bottom) and 2%/2mm (right bottom) criteria are reported. The gamma pass rates in this specific example were 87.5% (1%/1mm) and 98.1% (2%/2mm) for the analysis with D_T = 50%.

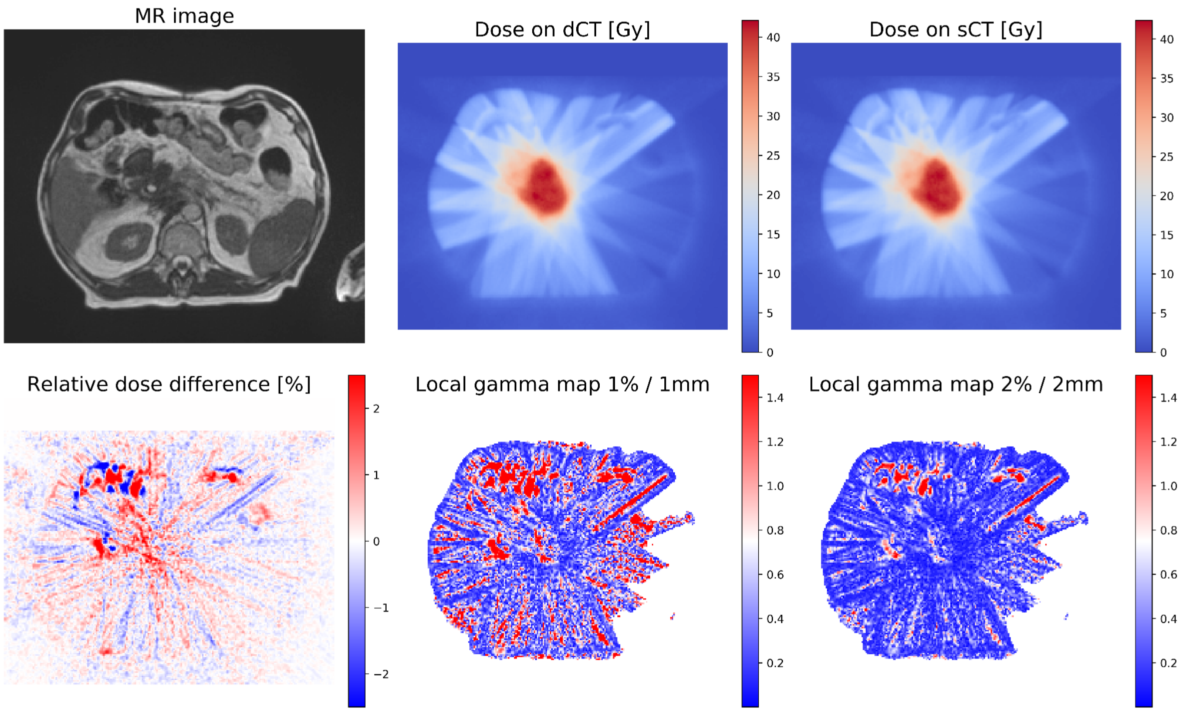

Supplementary Figure S5. Dose analysis for the outlier in the 1%/1mm gamma analysis with 90% threshold in Figure 3. The MR image (left top) is shown along with the dCT (centre top) and sCT (right top). The analysis of the voxel-by-voxel difference (left bottom) and gamma analysis with 1%/1mm (centre bottom) and 2%/2mm (right bottom) criteria are reported. The gamma pass rates in this specific example were 86.3% (1%/1mm) and 97.1% (2%/2mm) for the analysis with D_T = 50%.

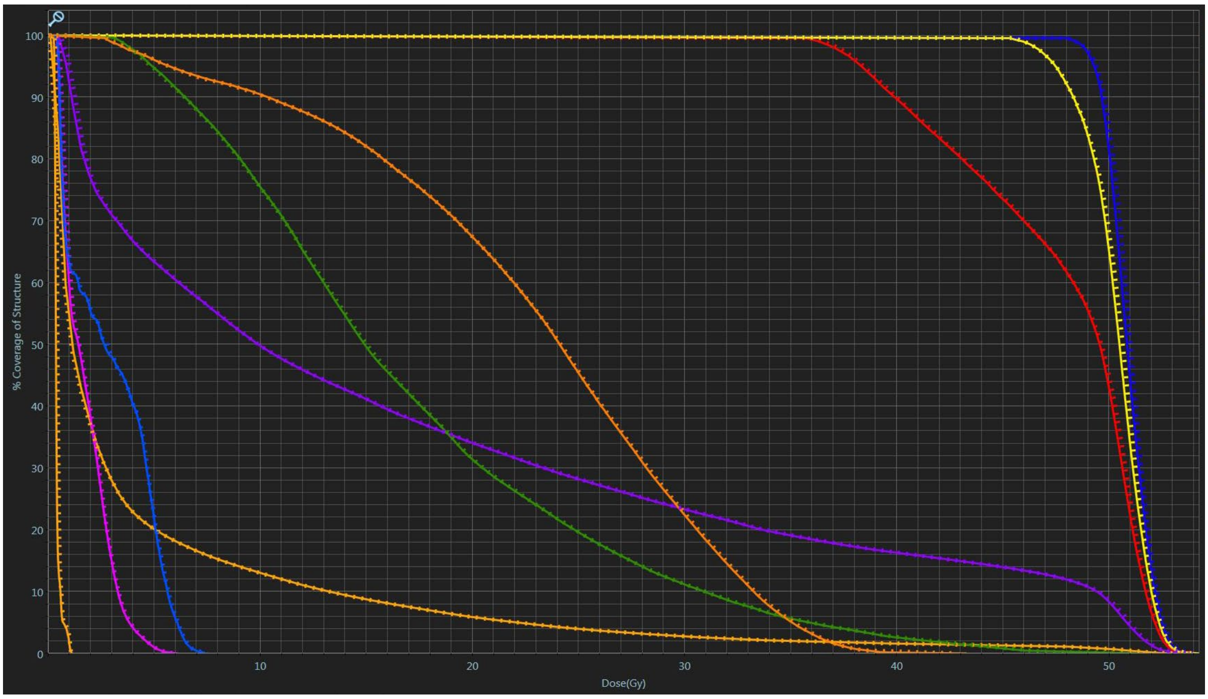

Supplementary Figure S6. DVH comparing dD (dashed) and sD (solid) for the liver metastasis segment VIII treatment reported in Figure 1a. The DVH report includes the GTV (blue), CTV (yellow), PTV (red), Ring2 (dark orange), Partial lung right (green), Liver (magenta), Bowel (light orange), Spinal cord (light blue).

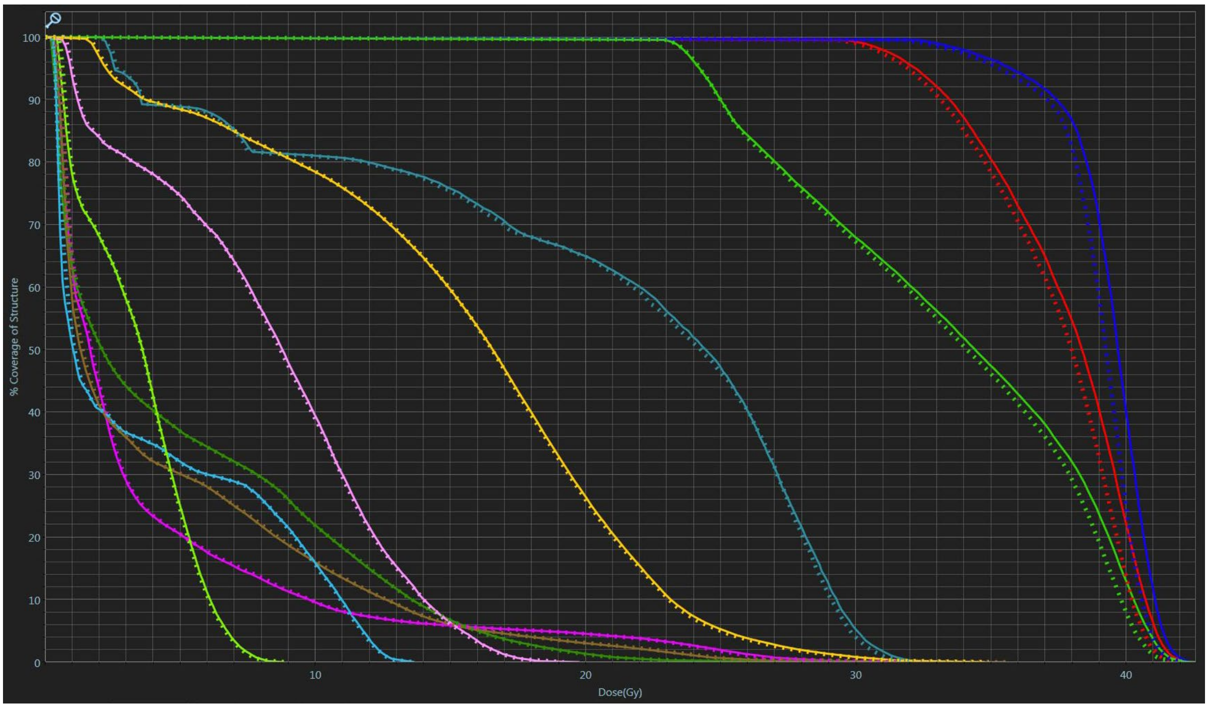

Supplementary Figure S7. DVH comparing dD (dashed) and sD (solid) for the outlier in the 1%/1mm gamma analysis with 50% threshold in Figure 3. The DVH report includes the GTV (blue), High dose PTV (red), Low dose PTV (bright green), Duodenum (turquoise), Ring2 (yellow), Kidney right (pink), Bowel (green), Spinal cord (light blue), Liver (brown), Stomach (magenta), Kidney left (lime green).

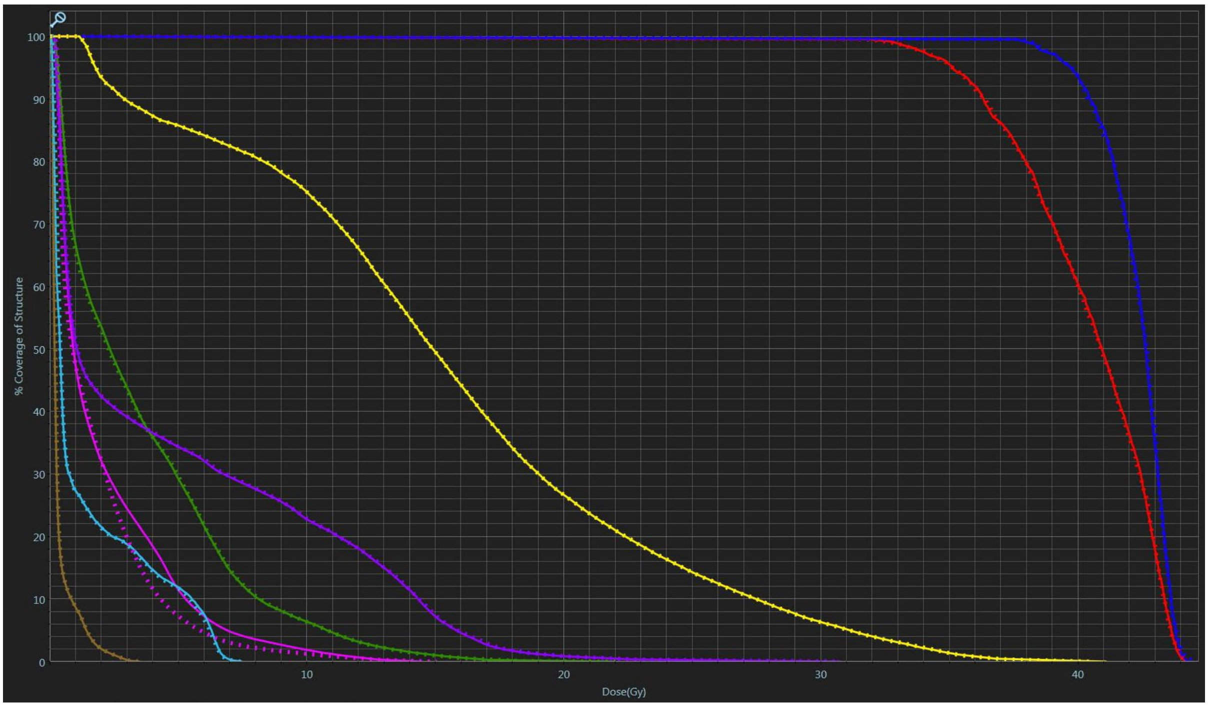

Supplementary Figure S8. DVH comparing dD (dashed) and sD (solid) for the adrenal gland metastasis treatment reported in Figure 1b. The DVH report includes the GTV (blue), PTV (red), Ring2 (yellow), Kidney left (violet), Bowel (green), Stomach (magenta), Spinal cord (light blue), Liver (brown).

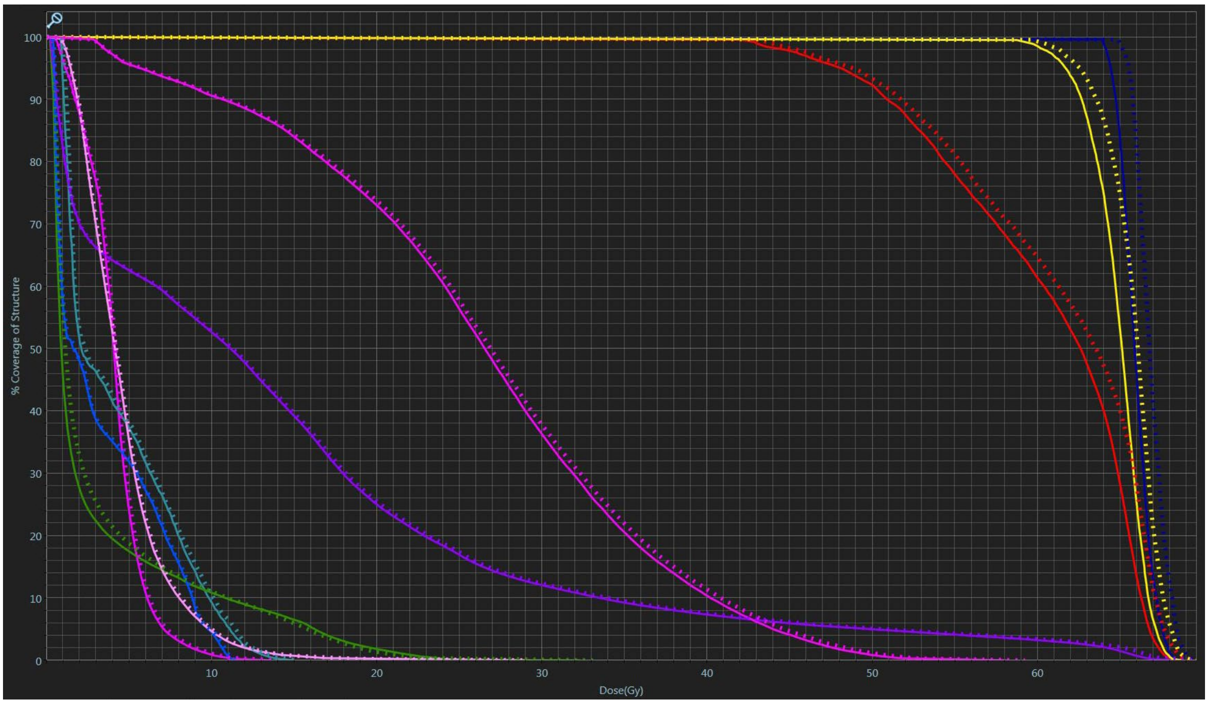

Supplementary Figure S9. DVH comparing dD (dashed) and sD (solid) for the outlier in the 1%/1mm gamma analysis with 90% threshold in Figure 3. The DVH report includes the GTV (blue), CTV (yellow), PTV (red), Ring2 (light purple), Liver (violet), Bowel (green), Duodenum (turquoise), Spinal cord (light blue), Heart (pink), Stomach (magenta)

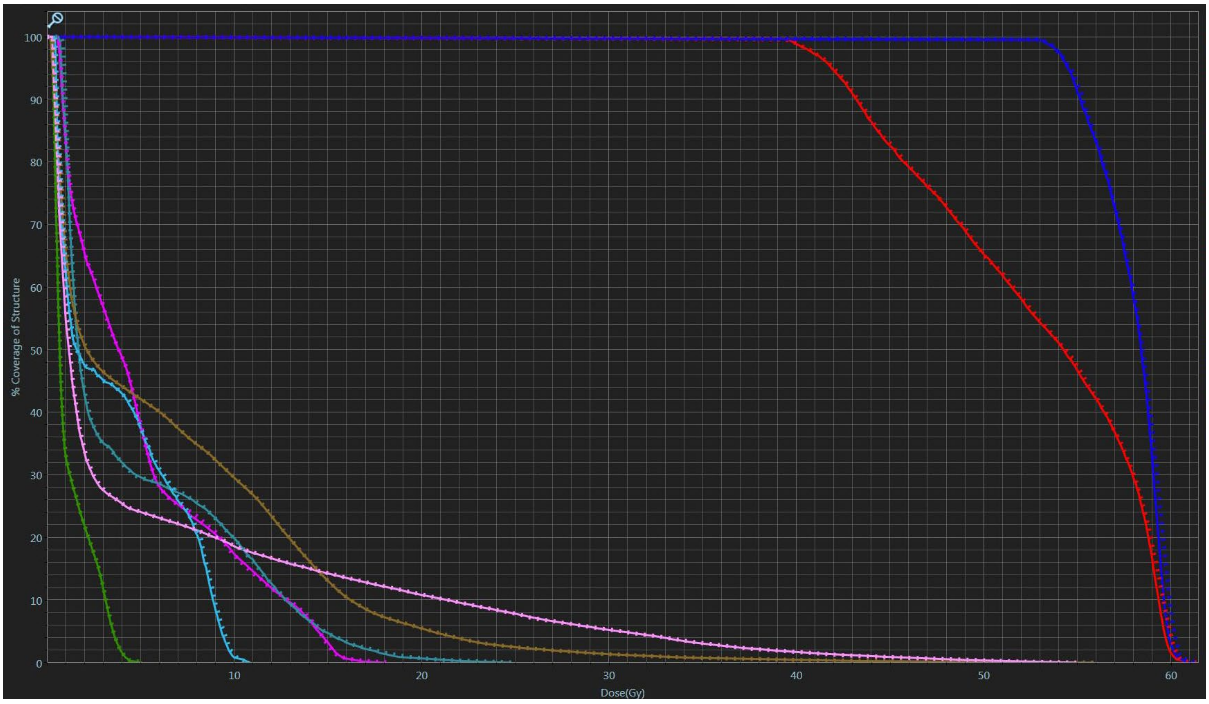

Supplementary Figure S10. DVH comparing dD (dashed) and sD (solid) for the dose comparison reported in Figure 4. The DVH report includes the GTV (blue), PTV (red), Kidney right (pink), Duodenum (turquoise), Stomach (magenta), Spinal cord (light blue), Liver (brown), Bowel (green)
